# Efficacy and tolerability Aripiprazole once-monthly long-acting injectable in schizophrenia. Two-injection start regimen

**DOI:** 10.1192/j.eurpsy.2022.2037

**Published:** 2022-09-01

**Authors:** S.L. Romero Guillena, B.O. Plasencia Garcia De Diego, F. Gotor Sanchez-Luengo, J. Gómez González

**Affiliations:** 1 UGC Salud Mental Virgen Macarena, , Psychiatry, seville, Spain; 2 Virgen del Rocio Hospital, Psychiatry, Seville, Spain

**Keywords:** Efficacy, schizophrénia, Aripiprazole once-monthly, Two-injection start regimen

## Abstract

**Introduction:**

Aripiprazole once-monthly is a long-acting intramuscular injectable formulation of aripiprazole. The starting dose can be administered by following one of two regimens: • One injection start: On the day of initiation, administer one injection of 400 mg Aripiprazole once monthly and continue treatment with 10 mg to 20 mg oral aripiprazole per day for 14 consecutive days • Two injection start (New regimen): On the day of initiation, administer two separate injections of 400 mg Aripiprazole once monthly at separate injection sites, along with one 20 mg dose of oral aripiprazole.

**Objectives:**

To assess the effectiveness and tolerability of Aripiprazole long-acting injectable (ALAI) in patients with schizophrenia. The starting dose was administered following the two injection start regimen

**Methods:**

Sample:10 patients with schizophrenia (DSM 5 criteria) who started treatment with ALAI. The starting dose was administered following the two injection start regimen. On a tri-monthly basis, the following evaluations were performed during a follow-up period of 6 months: The Clinical Global Impression-Schizophrenia scale (CGI-SCH), treatment adherence, the number of hospitalizations and Side effects reported

**Results:**

Mean variations from baseline scores at 6 months was (-1.1 ±0.89) on the GCI-SCH. The percentage of patients who remained free of admissions at the end of the 6 months was 90%. The rate of adherence to treatment after 6 months was 80%. The most frequent side effect was transient mild insomnia (20%) .

**Conclusions:**

Aripiprazole long-acting injectable (The starting dose was administered following the two injection start regimen) is effective, safe and well tolerated in clinical practice conditions

**Disclosure:**

No significant relationships.

